# African lineage 1a West Nile virus isolated from crocodiles exhibits low neuroinvasiveness in mice

**DOI:** 10.1099/jgv.0.002051

**Published:** 2024-11-26

**Authors:** Hiroko Kobayashi, Herman Chambaro, Koshiro Tabata, Takuma Ariizumi, Wallaya Phongphaew, Kunda Ndashe, Joseph Ndebe, Paul Fandamu, Shintaro Kobayashi, Naoto Ito, Michihito Sasaki, Bernard M. Hang’ombe, Edgar Simulundu, Yasuko Orba, Hirofumi Sawa

**Affiliations:** 1 Division of Molecular Pathobiology, International Institute for Zoonosis Control, Hokkaido University, Sapporo, Japan; 2 Department of Veterinary Services, Ministry of Fisheries and Livestock, Lusaka, Zambia; 3 Institute for Vaccine Research and Development, Hokkaido University, Sapporo, Japan; 4 Department of Pathology, Faculty of Veterinary Medicine, Kasetsart University, Bangkok, Thailand; 5 Department of Disease Control, School of Veterinary Medicine, University of Zambia, Lusaka, Zambia; 6 Laboratory of Public Health, Faculty of Veterinary Medicine, Hokkaido University, Sapporo, Japan; 7 Laboratory of Zoonotic Diseases, Faculty of Applied Biological Sciences, Gifu University, Gifu, Japan; 8 Department of Para-clinical Studies, School of Veterinary Medicine, University of Zambia, Lusaka, Zambia; 9 Africa Centre of Excellence for Infectious Diseases of Humans and Animals, Lusaka, Zambia; 10 Macha Research Trust, Choma, Zambia; 11 International Collaboration Unit, International Institute for Zoonosis Control, Hokkaido University, Sapporo, Japan; 12 One Health Research Center, Hokkaido University, Sapporo, Japan; 13 Global Virus Network, Baltimore, Maryland, USA

**Keywords:** crocodile, flavivirus, pathogenicity in mice, virus isolation, West Nile virus

## Abstract

West Nile virus (WNV) is a mosquito-borne flavivirus that causes encephalitis in humans and infects crocodiles, resulting in rashes and neurological signs. In Zambia, two distinct lineages of WNV have been detected in neighbouring areas: lineage 2 in mosquitoes and lineage 1a in farmed crocodiles. Considering the risk of direct or vector-mediated WNV transmission from crocodiles to mammals, it is necessary to elucidate the pathogenicity of WNV strains derived from crocodiles. In this study, WNV was successfully isolated from naturally infected farmed crocodiles (Croc110/2019/1/ZM, Croc110). We then investigated its proliferation and pathogenicity in mice in comparison with a WNV isolate from mosquitoes in Zambia (Zmq16) and two reference strains, including one highly pathogenic (NY99) and one low pathogenic (Eg101) strain. Although viral proliferation in Vero and mammalian neuronal cells was comparable among the strains, Croc110 exhibited low cell-to-cell transmission efficiency. *In vivo*, more than 70% of mice (C57BL/6) intracerebrally inoculated with Croc110 displayed neurological signs, and Croc110-infected mice exhibited similarly high mortality rates as NY99- and Zmq16-infected mice. Meanwhile, comparable virus growth was observed among the strains in the brain. However, the virulence of Croc110 was significantly lower than that of Zmq16 and NY99 following intradermal (ID) and intraperitoneal inoculation. Consistently, Croc110 displayed lower growth than Zmq16 and NY99 in the brain and peripheral tissues after ID inoculation. Our study revealed that the crocodile-derived WNV strain is less neuroinvasive in mice, and it exhibits distinct pathogenicity from the highly pathogenic mosquito-derived WNV strain circulating in Zambia.

## Introduction

West Nile virus (WNV) is a single-stranded RNA virus belonging to the genus *Orthoflavivirus* within the family Flaviviridae. This virus circulates between mosquitoes, especially *Culex* species, and wild birds in the field, and it can be transmitted to humans and animals through mosquito bites [1,2]. In humans, ~80% of infected individuals are asymptomatic, whereas the rest may report fever, headache, fatigue and gastrointestinal signs. In severe cases, WNV infection causes neuroinvasive diseases, such as encephalitis and meningitis [3,5].

WNV infection has been previously reported in farmed crocodilians (i.e. *Crocodylus niloticus*, *Crocodylus moreletii* and *Alligator mississippiensis*) [6,10]. Crocodiles infected with WNV can develop neurological disorders such as swimming in circles and lymphohistiocytic proliferative cutaneous lesions, also called ‘pix’, leading to significantly worse commercial skin quality [10,11]. Previous reports suggested that WNV can be transmitted to crocodiles through mosquito bites, consumption of infected birds and horizontal transmission through water in the habitat [12,13].

Although no human clinical case of West Nile fever has been reported in Zambia, our previous studies revealed the circulation of two different WNV lineages in Zambia [6,14]. The linage 2 WNV strain Zmq16m11 (Zmq16) was isolated from *Culex* mosquitoes captured in the Western Province [14]. On the contrary, a lineage 1 WNV was detected by reverse transcription-polymerase chain reaction (RT-PCR) in whole-blood samples from farmed crocodiles in the Southern Province of Zambia, and its genomic sequence was reported as Croc110/2019/ZM [6]. In this study, we succeeded in isolating this virus from crocodiles.

The pathogenicity of WNV can be assessed in rodent models. Immunocompetent mouse models, such as C57BL/6 and C3H/HeN, have been extensively used. The mouse models exhibit lethal neurological signs following intraperitoneal (IP) or intradermal (ID) WNV infection, providing insight into the characterization of WNV pathogenicity [5,15,17]. In this study, to estimate the pathogenicity of two Zambian WNV strains in mammals, we investigated the clinical manifestations and viral replication of the two WNV isolates using the C57BL/6 mouse model. In comparison with two prototype strains, one highly pathogenic (NY99) and one low pathogenic (Eg101) strain [18,20], the crocodile-derived isolate (Croc110) exhibited low pathogenicity, whereas the mosquito-derived isolate (Zmq16) was highly pathogenic in mice.

## Methods

### Cells and viruses

Unless otherwise stated, the cells were cultured in a medium supplemented with 10% FBS and an antibiotic mixture (penicillin, 100 unit ml^−1^; streptomycin, 100 µg ml^−1^). Mosquito-derived C6/36 cells were cultured in Eagle’s Minimum Essential Medium (MEM, Wako, Osaka, Japan) supplemented with non-essential amino acid solution. Vero 9013 cells (Vero cells; JGRB, Ibaraki, Japan), SH-SY5Y human neuroblastoma cells and mouse neuroblastoma (NA) cells were cultured in Dulbecco’s Modified Eagle’s Medium (DMEM, Wako), DMEM F12 (Thermo Fisher Scientific, Waltham, MA, USA) and MEM, respectively. MEM containing 2% FBS and an antibiotic mixture was used during viral infection. C6/36 cells were incubated at 28 ℃, and the other cell lines were incubated at 37 °C, both in the presence of 5% CO_2_. The NY99 (accession no. AB185914), Eg101 (accession no. AF260968), Zmq16m11 (accession no. LC318700) and Croc110 were propagated in C6/36 cells at 28 ℃.

### Virus titration

Virus titration was assessed in Vero cells using the plaque assay. Briefly, Vero cells were cultured in six-well plates and infected with tenfold serially diluted WNV. After 1 h of incubation with agitation every 10 min, the supernatant was removed, and the cells were overlaid with MEM supplemented with 2% FBS, 1% methylcellulose and 1% antibiotic mixture, and incubated at 37 ℃. At 4 days post-infection (dpi), Vero cells were formalin-fixed and stained with 1% crystal violet. The plaqu-forming unit was expressed as p.f.u. and finally used to calculate the viral m.o.i., which was defined as 1 p.f.u. per cell in this study.

### Virus isolation

The Croc110 strain, previously reported by Simulundu *et al.*, was isolated from crocodile blood using a slightly modified version of the original method [6,21]. Blood (*n* = 22) was collected in EDTA tubes from juvenile crocodiles via the post-occipital sinus of the spinal vein. RNA was extracted using the QIAamp viral RNA mini Kit (QIAGEN, Venlo, Netherlands) according to the manufacturer’s instructions. WNV RNA was screened using an in-house primer pair and probe (probe, 5′-FAM-GATCTCGATGTCTAAGAAACCAGGAGG-3′-minor groove binder (MGB)-Eclipse; F, 5′-GCGAGCTGTTTCTTRGCAC-3′; R, 5′- AGRCTCARCATWGCCCTCTT-3′) in the ABI Step One real-time PCR-system (ABI, Warrington, UK) using the Thunderbird probe one-step qRT-PCR kit (TOYOBO, Osaka, Japan). Four samples were used to identify the sequence in which the *C*_t_ value was lower than 37 on reverse transcription-quantititative polymerase chain reaction (RT-qPCR) for each sample. The partial sequences of the structural protein-coding region were identified by conventional PCR and Sanger sequencing with specific primers [6], and a 100% match was observed in all samples. Each blood sample was diluted 100-fold in MEM supplemented with non-essential amino acid solution and 2% FBS and filtrated twice. The filtrate was serially diluted (1 : 100–1 : 10 000) and inoculated onto a C6/36 cell monolayer under biosafety level 3 containment. The cytopathic effects (CPEs) of cells inoculated with filtrates were examined each day for 7 days. One of the four samples was used as the Croc110 isolate.

### Electron microscopic examination of the Croc110 isolate

The supernatant from C6/36 cells inoculated with Croc110 (m.o.i. of 0.01) for 5 days was collected and fixed with paraformaldehyde (final concentration of 2.5%) overnight at 4 °C. After fixation, the supernatant was centrifuged to remove the cellular debris, filtrated using 0.22 µm filters and then ultracentrifuged at 110 000 ***g*** on a 20% sucrose cushion at 4 ℃ for 3 h. The precipitant after ultracentrifugation was suspended in PBS overnight at 4 ℃. Dissolved samples (20 µl) were spotted onto elastic carbon grids (Okenshoji Co., Ltd., Tokyo, Japan) for 5 min. Thereafter, the grids were washed with PBS three times. The samples on the grids were stained with 2% phosphotungstic acid (pH 5.8) and observed using a transmission electron microscope (TEM HT7800, HITACHI, Tokyo, Japan).

### *In vitro* viral growth assay

Croc110 (m.o.i. of 0.01) was inoculated into C6/36 cells and NY99, Eg101, Croc110 and Zmq16 (m.o.i. of 0.001) were inoculated into Vero, NA and SH-SY5Y cells in 24-well plates for 1 h. Then, the cells were washed with PBS and maintained in a medium. The supernatant was collected from each cell line at 1, 2, 3 and 4 dpi. Virus titration in the supernatant was assessed using the TCID_50_ assay with Vero cells. Briefly, WNV-inoculated cell supernatants were serially diluted tenfold in 96-well plates, and this dilution was repeated four times in each sample. The diluted samples were inoculated into Vero cells in 96-well plates. The CPEs in each well were monitored for 7 days. The Reed and Muench method was used to calculate the viral titres as TCID_50_ ml^−1^ [22]. Three independent experiments were performed under identical conditions. The mean of three viral titres at each time point is presented in the Results.

### Immunofluorescent staining and focus-forming assay

Croc110 (m.o.i. of 0.1) was inoculated into C6/36 cells in six-well plates and incubated with agitation at 28 ℃ for 1 h. After infection, cells were overlaid with MEM supplemented with 2% FBS, 1% methylcellulose and 1% antibiotic mixture, and incubated at 28 ℃. At 3 dpi, cells were fixed with formalin, permeabilized with 0.2% Triton X-100 and blocked with 1% BSA in PBS. Cells were stained with an anti-flavivirus NS1 antibody (4G4) (1 : 500, Mozzy Mabs, kindly provided by Professor Roy Hall, University of Queensland, Australia) [23,24] for 1 h, stained with Alexa 488-labelled anti-mouse IgG (1 : 1,000, Invitrogen, Carlsbad, CA, USA) and counterstained with Hoechst 33342 for 1 h. Fluorescent images were evaluated using an Olympus IX-73 microscope (Olympus, Tokyo, Japan).

To analyse cell-to-cell viral propagation by the focus-forming assay, each WNV strain was inoculated into Vero, NA and SH-SY5Y cells in six-well plates as previously described. Following incubation (Vero, 36 h; NA, 72 h; SH-SY5Y, 30 h), cells were fixed in formalin and stained with 4G4 antibody. The stained focus area (*n* = 50) was measured using Cell Sens Software (Evident, Tokyo, Japan).

### Whole-genome sequencing and phylogenetic analysis

Viral RNA was extracted from Croc110-infected cell supernatant using the High Pure Viral RNA Kit (Roche, Basel, Switzerland) and reverse-transcribed using the PrimeScript Double Standard cDNA Synthesis Kit (Takara, Shiga, Japan). DNA libraries were prepared using the Nextera XT DNA Library Preparation Kit (Illumina, San Diego, CA, USA) and sequenced using the Illumina iSeq platform (Illumina). Reads were processed and mapped to a reference strain (accession number: KU588135.1) using the CLC Genomics Workbench (QIAGEN). The mapped reads were extracted and used for *de novo* assembly to determine the full-length Croc110 sequence. Since some sequences in the 3′-untranslated region (UTR) could not be determined, Sanger sequencing of the 3′-UTR region was performed using the primers designed on the basis of a lineage 1a strain (accession no. AF404757; F, 5′-GAGGACATGCTGGAGGTTTGGAAC-3′; R, 5′-AGATCCTGTGTTCTCGCACCACC-3′). To confirm the absence of the genomes of other pathogens in the isolated virus sample, unmapped reads were also used for *de novo* assembly and contigs more than 500 nucleotides in length were extracted and analysed by Multi-blast (https://ftp.ncbi.nlm.nih.gov/blast/executables/blast+/LATEST/).https://ftp.ncbi.nlm.nih.gov/blast/executables/blast+/LATEST/)

Phylogenetic analysis of the polyprotein aa sequences of WNV was performed by the maximum likelihood method. Each sequence used in the phylogenetic analysis was obtained from GenBank and aligned with ClustalW [25]. The evolutionary history was inferred by the maximum likelihood method and JTT+G+I model using mega11 [26,27].

### Animal experiments

Groups (*n* = 6) of female C57BL/6 J mice (6 weeks old) were intracerebrally (10 p.f.u. 20 µl^−1^), intraperitoneally (100 p.f.u. 100 µl^−1^) or intradermally (100 p.f.u. 50 µl^−1^) infected with NY99, Eg101, Croc110 or Zmq16, or mock-infected with virus-free medium. The survival rate and body weight changes of mice were monitored for 13 days after intracerebral (IC) inoculation and for 22 days after ID or IP inoculation. Experiments were replicated twice. Clinical signs were monitored every day and evaluated using a clinical score as follows: 0, healthy; 1, 7.5% body weight loss versus the maximum body weight; 2, unsteady gait; 3, kinetic tremors and severe ataxia; 4, paralysis and 5, death or humane endpoint euthanasia. The criteria for the humane endpoint were 25% body weight loss versus maximum body weight and a loss of spontaneous drinking or feeding.

The serum samples of surviving mice inoculated intradermally or intraperitoneally were collected for further neutralizing antibody testing. After heat inactivation of the serum at 56 °C for 30 min, samples were first diluted fivefold and then diluted serially twofold in the medium. Diluted sera were mixed 1 : 1 with 100 TCID_50_ of each virus strain and incubated at 37 °C for 1 h. Following incubation, mixed samples were inoculated into Vero cells and observed for 7 days. Serum concentrations that inhibited CPEs by 50% were taken as neutralizing antibody titres.

To evaluate viral RNA levels in tissues, mice intradermally or intracerebrally inoculated with WNV were sacrificed via humane euthanasia under deep anaesthesia. Serum, spleen, brain and small intestine samples were collected from intradermally infected mice at 3, 6 and 9 dpi, and the brain samples were collected from intracerebrally infected mice at 2, 4 and 6 dpi. Half of the brain or intestine and the entire spleen were used and homogenized in PBS (800 µl for brain samples, 500 µl for spleen samples and 3 ml for small intestine samples). The clarified supernatant (100 µl) and serum samples were used for RNA extraction. Viral RNA levels in whole tissue were determined by RT-qPCR as previously mentioned, corrected according to the tissue volume, and expressed as p.f.u. in whole tissues using a calibration curve generated using a previously titrated sample.

### Statistical analysis

All statistical analyses were performed using GraphPad Prism 10.1.1. Mann–Whitney *U* test was performed to compare the viral RNA amounts of Croc110 and each of the other strains. For the comparison of three or more groups, one-way ANOVA with Dunnett’s test was used. *P* < 0.05 was considered as statistically significant.

## Results

### Virus isolation and phylogenetic analysis of Croc110/2019/1/ZM

**Fig. 1.**
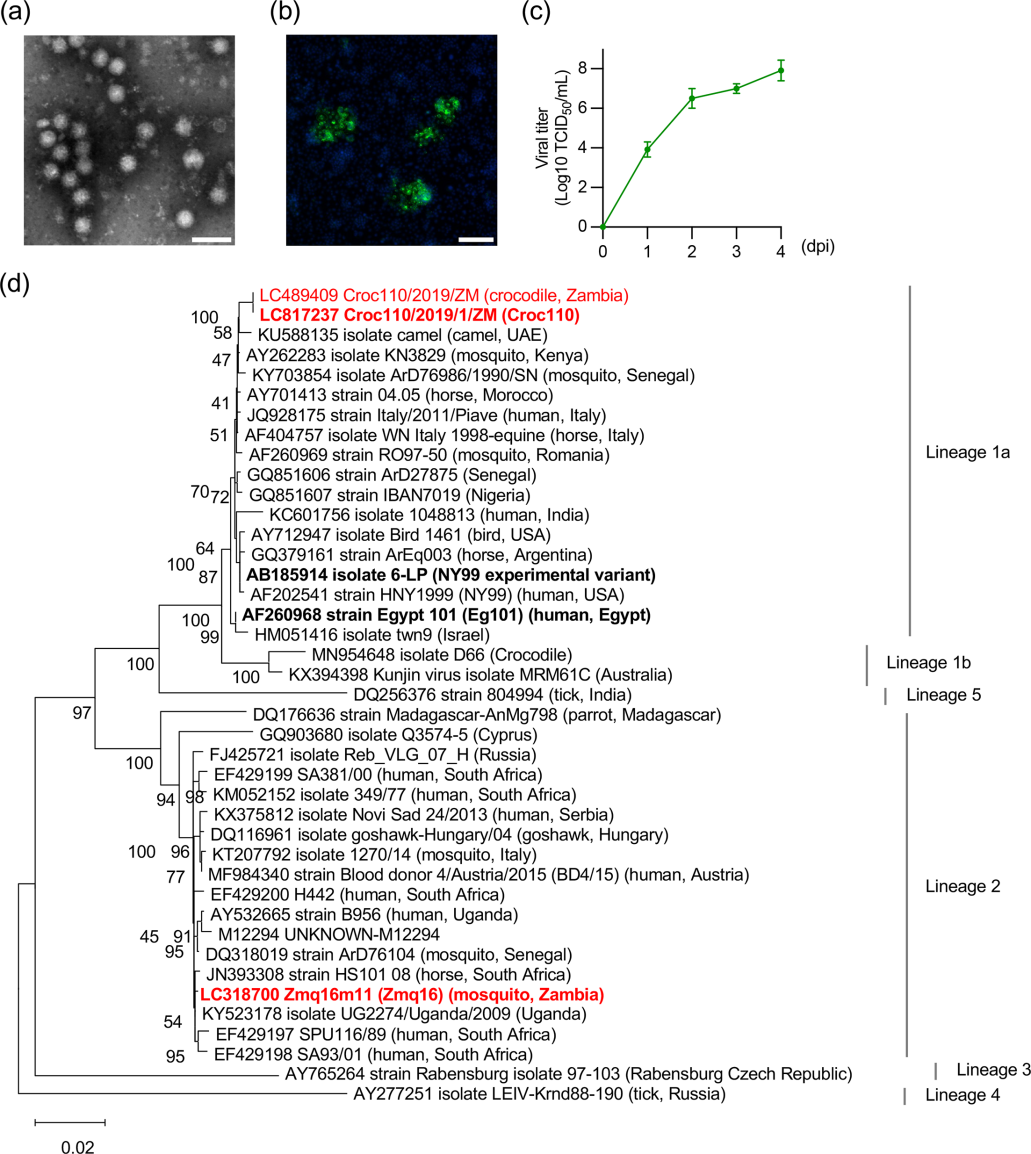
Characterization of the WNV Croc110 strain isolated from crocodiles. (**a**) Electron microscopic image of negatively stained Croc110 particles. Scale bar, 100 nm. (**b**) Focus formation by Croc110 in C6/36 cells. Cells were infected with Croc110 at an m.o.i. of 0.01 and stained with anti-flavivirus NS1 antibody at 4 dpi (green). Cell nuclei were counterstained with Hoechst (blue). Scale bar, 100 µm. (**c**) The viral growth kinetics of Croc110 in C6/36 cells. The viral titre in the supernatant was determined by the TCID_50_ assay using Vero cells. The log-transformed values of viral titre are presented on a log_10_ scale as the mean ± sd of three independent experiments. (**d**) Phylogenetic tree of Croc110 and other representative WNV strains. Phylogenetic analysis was performed using the full-length WNV polyprotein aa sequences by the maximum likelihood method with 1000 bootstrap replicates. The tree is drawn to scale, with branch lengths measured in the number of substitutions per site. The isolated Croc110, Croc110/2019/1/ZM, previously identified Croc110/2019/ZM and Zmq16 are indicated in red text. The WNV strains used in this study are denoted in bold.

We attempted to isolate WNV from the blood samples of diseased juvenile crocodiles that showed clinical signs, including anorexia, weakness, swimming in circles, bloody diarrhoea and scoliosis [6]. Of the 16 samples positive for WNV (16/22), four samples with *C*_t_ values lower than 37 were used for virus isolation. CPEs in C6/36 cells were evident at 4 dpi in all the inoculated samples. Negative-staining electron microscopy of the cell supernatant identified virus particles of ~50 nm in diameter, consistent with flaviviruses (Fig. 1a). In C6/36 cells, foci positive for WNV antigen were observed at 4 dpi, and the viral titre in the supernatant increased up to 1×10^7^ TCID_50_ ml^−1^ within 2 dpi (Fig. 1b, c). Next-generation sequencing of total RNA from the supernatant of the virus-infected cells and Sanger sequencing of the 3'-UTR were performed to determine the entire genome of WNV. Compared to the previously reported polyprotein sequence of Croc110/2019/ZM (accession number: LC489409) [6], which was obtained by RT-PCR from one of the blood samples used in this study, the genome sequence of our isolated WNV (Croc110/2019/1/ZM, Croc110, accession number: LC817237) had four nucleotide substitutions. Nonetheless, our isolate and Croc110/2019/ZM were 100% identical at the aa level. Phylogenetic analysis based on the aa sequence of WNV polyproteins illustrated that Croc110 belongs to lineage 1a, and it is most closely related to a WNV strain detected from a camel in the United Arab Emirates (accession no. KU588135) [28] (Fig. 1d). Although no whole-genome sequence data have been deposited for other WNV isolates from crocodilians, our molecular phylogenetic data suggest that WNV circulating in crocodiles does not form a unique lineage. To further investigate aa substitutions characteristic of the Croc110 strain, we compared sequences between Croc110 and representative lineage 1a and lineage 2 WNVs (Table 1). In the coding sequences, 13 aa differences were detected in the Croc110 strain compared with the other strains, and notably, four unique substitutions were found in NS4B.

**Table 1.** The aa differences characteristic of Croc110 compared to other WNV strains

	Protein	prM	E		NS1	NS3			NS4B				NS5	
	aa Position	35	7	44	96	157	334	368	8	31	72	102	42	791
Lineage 1a	Croc110LC817237	V	G	R	L	A	F	T	R	G	V	S	Y	D
	Camel KU588135	I	S	K	M	P	S	M	K	E	I	C	H	N
	KN3829 AY262283	I	S	K	M	P	S	M	K	E	I	C	H	N
	NY99 AF202541	I	S	K	M	P	S	M	K	E	I	C	H	N
	Eg101 EU081844	I	S	K	M	P	S	M	K	E	I	C	H	N
Lineage 2	SA93/01 EF429198	I	S	K	M	P	S	M	K	S	I	C	H	N
	Zmq16 LC318700	I	S	K	M	P	S	M	K	S	I	C	H	N

### Comparative analysis of the *in vitro* growth of WNV isolates

**Fig. 2.**
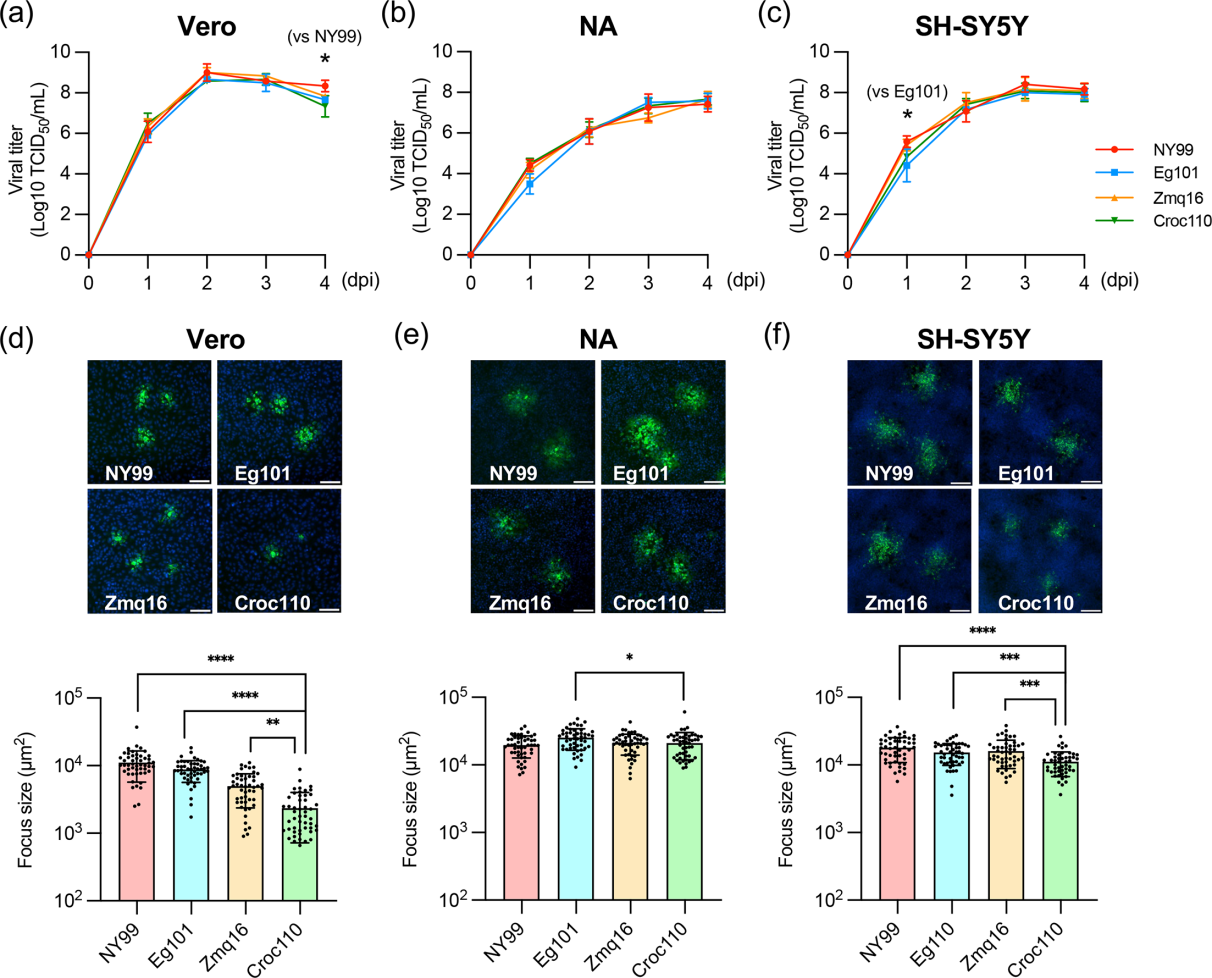
Comparative analysis of viral growth kinetics *in vitro* proliferation of Croc110, Zmq16, NY99 and Eg101 in Vero (**a**), NA (**b**) and SH-SY5Y cells (**c**). The viral titre in the supernatant was determined by the TCID_50_ assay using Vero cells. The log-transformed values of viral titre are presented on a log_10_ scale as the mean±sd of three independent experiments. Cell-to-cell viral propagation in Vero (**d**), NA (**e**) and SH-SY5Y cells (**f**). Focus formation was visualized with anti-flavivirus NS1 antibody (green) and nuclear staining (blue) at 36 h post-infection in Vero cells, 3 dpi in NA cells and 30 h post-infection in SH-SY5Y cells. Representative focus images of each WNV strain are presented. Scale bar, 100 µm. Foci (*n*=50) were randomly selected to measure the focus area. The values are presented as the mean±sd of 50 foci from one experiment. Statistical analysis was performed by one-way ANOVA and Dunnett’s multiple-comparison test in comparison to the result for Croc110 (*P<0.05; **P<0.01; ***P<0.001and ****P<0.0001).

The propagation of Croc110 in mammalian cells was compared with that of another Zambian strain (Zmq16) and lineage 1 reference strains (NY99 and Eg101). First, we analysed viral growth kinetics in Vero cells, mouse neuroblastoma-derived NA cells and human neuroblastoma-derived SH-SY5Y cells (Fig. 2a–c). There was no distinct difference in the titres between Croc110 and other WNV isolates in all the cell lines. Conversely, Croc110 formed significantly smaller foci in Vero and SH-SY5Y cells than the other strains in focus-forming assay by NS1 staining (Fig. 2d, f). In NA cells, only Eg101 had a significantly larger focus size than Croc110 (Fig. 2e). Similar results were confirmed by assays using E protein staining, as staining of secreted NS1 may affect focus size. These results indicate that the cell-to-cell infection efficiency of Croc110 is lower than that of the other strains.

### Differences in the pathogenicity of WNV isolates in C57BL/6 mice

**Fig. 3.**
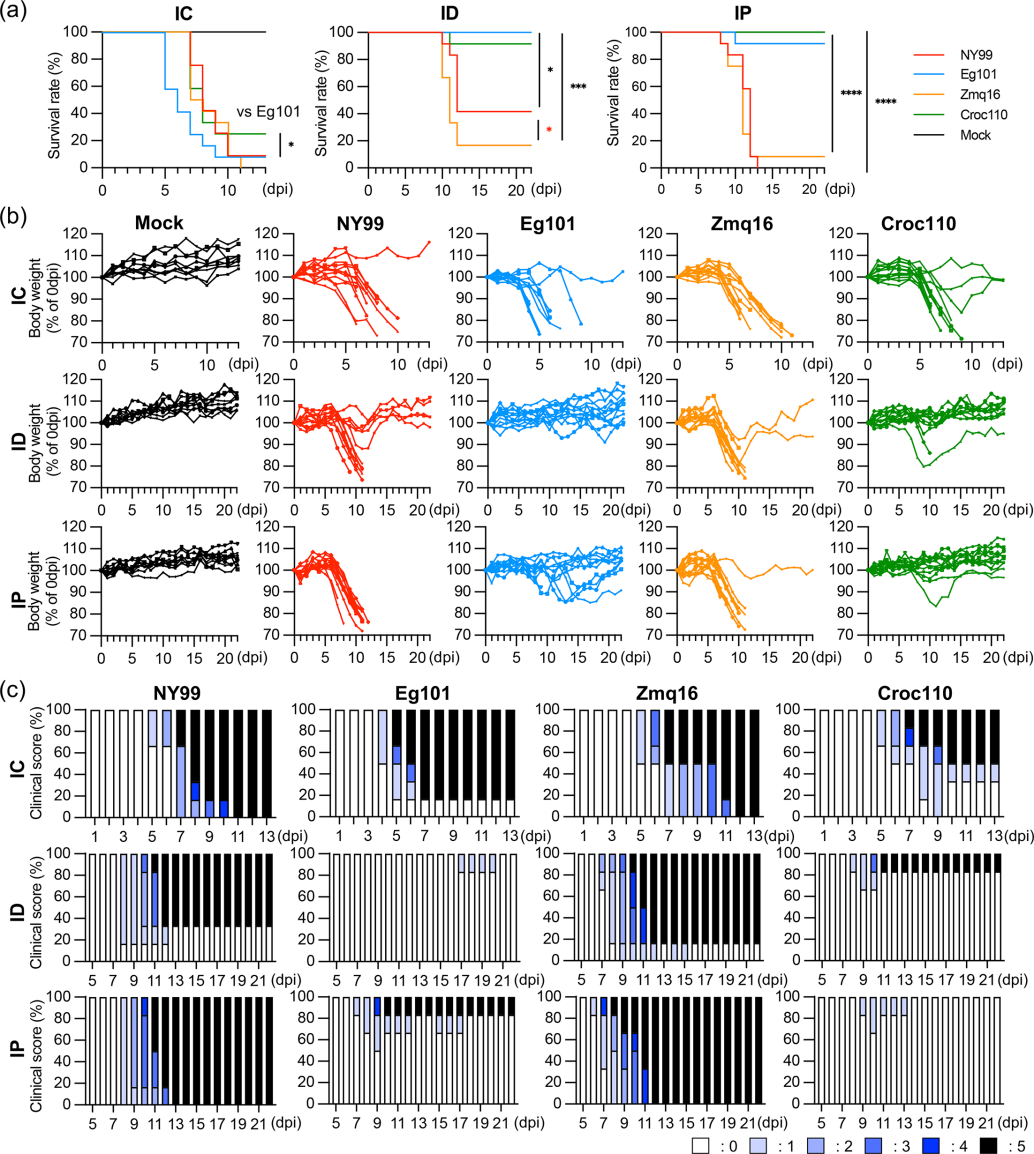
Differences in the pathogenicity of WNV isolates in C57BL/6 mice. C57BL/6 mice were infected with Croc110, Zmq16, NY99 or Eg101 or mock-infected intracerebrally (IC), intradermally (ID) or intraperitoneally (IP). Survival rates (**a**), body weight changes (**b**) and clinical signs (**c**) were monitored every day. A total of 12 (for each strain) or 9 mice (mock infection) were used in this study (**a, b**). Body weight at 0 dpi was set as 100 % (**b**). Clinical signs were monitored (*n*=6) and scored as follows: 0, healthy; 1, body weight loss; 2, unsteady gait; 3, kinetic tremors and severe ataxia; 4, paralysis and 5, death. The percentage of each clinical score is presented as a bar graph. (**c**). Statistical analysis was performed using one-way ANOVA and the log-rank test in comparison to the result for Croc110 (*P<0.05; ***P<0.001 and ****P<0.0001). The result of statistical analysis between Zmq16 and NY99 is indicated by red letters.

To compare the pathogenicity of Zambian WNV isolates in mice with that of NY99 and Eg101, each WNV was intracerebrally, intradermally or intraperitoneally inoculated into C57BL/6 mice. We performed ID inoculation into the skin of the auricle to mimic the transmission by mosquito bites [29]. In the case of IC inoculation, the survival rate of mice inoculated with Croc110 was 25%, which was slightly higher than that for mice inoculated with Eg101, and the body weight loss was observed for all strains (Fig. 3a, b). However, the survival rates of mice inoculated with Croc110 were 91.7 and 100% following ID and IP inoculation, respectively, similar to those observed for Eg101 (100% following ID inoculation and 91.7% following IP inoculation; Fig. 3a). High mortality rates were observed in mice inoculated with the Zmq16 and NY99 strains (Fig. 3a). Mice that were intradermally or intraperitoneally inoculated with Croc110 displayed no obvious body weight loss, excluding a few mice that succumbed or recovered after 10 dpi (Fig. 3b). In addition, the survival rate of mice intradermally inoculated with Zmq16 (16.7%) was lower than that of mice inoculated with NY99 (41.7%).

Sera from surviving mice inoculated intradermally or intraperitoneally were collected and assayed for neutralizing antibodies (Fig. S1, available in the online version of this article). Neutralizing antibodies were detected in all surviving mice, indicating that infections occurred in surviving mice. Clinical signs such as body weight loss, lethargy, unsteady gait, kinetic tremor, severe ataxia and paralysis were scored on a 5-point scale [30] (Fig. 3c). Signs were consistent with the body weight changes, with neurological signs being prominent in fatal cases. The earliest appearance of neurological signs was found in mice intradermally and intraperitoneally inoculated with Zmq16. These results suggest that the pathogenicity of Croc110 was comparable to that of the low pathogenic strain Eg101, whereas Zmq16 was highly pathogenic following peripheral inoculation.

### Replication of WNV isolates in tissues

**Fig. 4.**
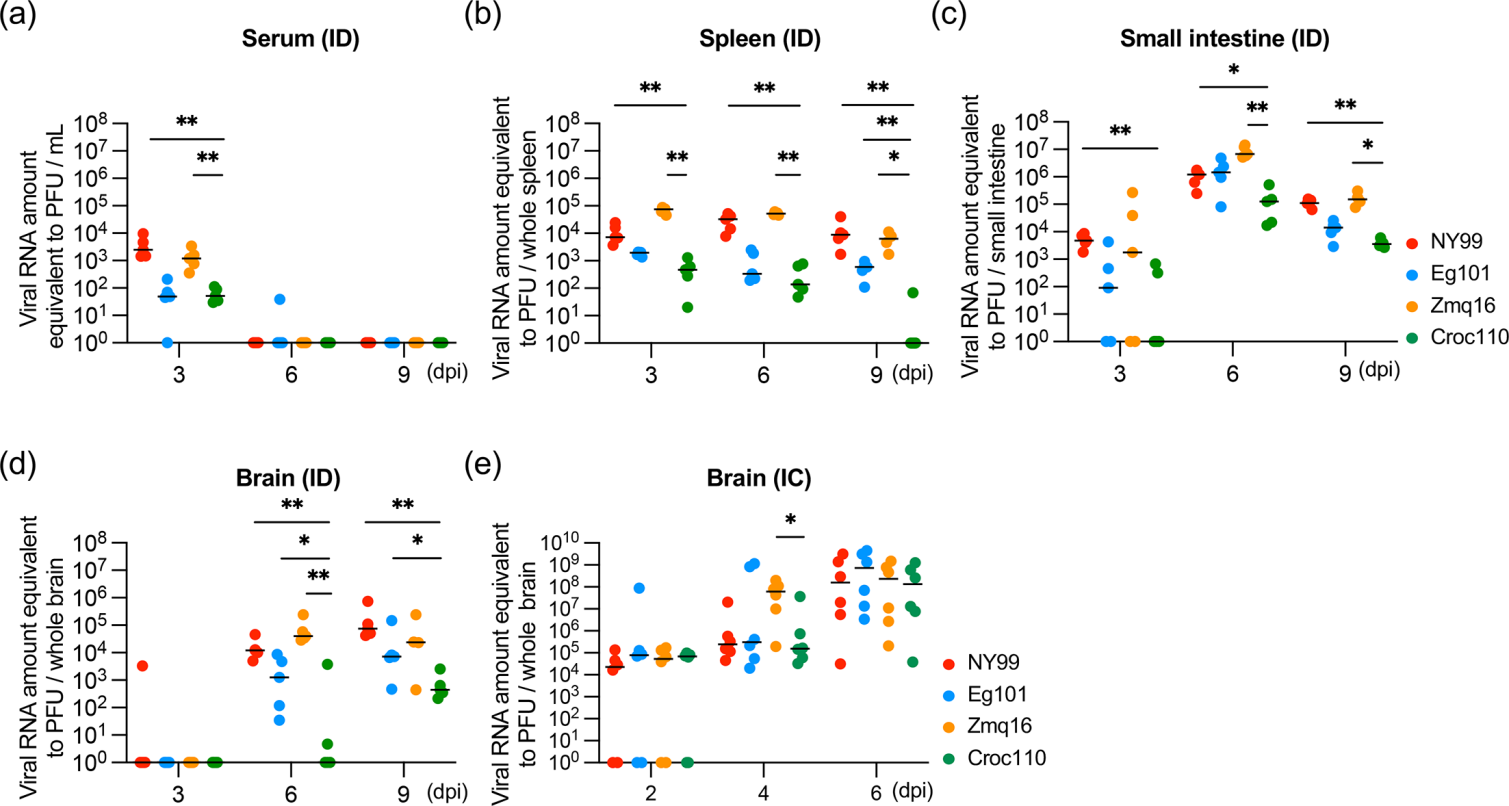
Propagation of each WNV strain in the brain and peripheral tissues. The viral RNA levels in the serum (**a**), spleen (**b**), small intestine (**c**) and brain (**d**) of C57BL/6 mice intradermally (ID) infected with each WNV strain (*n*=5) and the RNA level in the brain following intracerebral (IC) inoculation (*n*=6) (**e**). In the Zmq16-infected group at 9 dpi, four mice were used for the analysis because one mouse died before analysis (**a–d**). The amount of viral RNA was quantified by RT-qPCR and presented as p.f.u. equivalents, i.e. corrected as the amount in whole tissue samples. Individual values were presented as each plot, with black lines indicating the median for each group. Statistical analysis was performed using the Mann–Whitney *U* test in comparison to the result for Croc110 (*P<0.05; **P<0.01; ***P<0.001 and ****P<0.0001).

The viral replication was next evaluated in tissues from C57BL/6 mice intradermally inoculated with WNV (Fig. 4a–d) and in brain tissue following IC inoculation (Fig. 4e) using an RT-qPCR assay. When viral RNA levels in sera and peripheral tissues were analysed, an increase in viral RNA was observed in the small intestine from 3 to 6 dpi for all strains, indicating productive infection in the intestine following intradermal inoculation. The RNA levels of Croc110 were significantly lower than those of the highly pathogenic strains NY99 and Zmq16 in the serum, spleen, small intestine and brain (Fig. 4a–d). In the brain, the viral RNA of Zmq16, NY99 and Eg101 was detectable at 6 dpi, whereas Croc110 RNA was below the detection limit in the majority of brain samples (Fig. 4d). By contrast, IC inoculation resulted in comparable viral growth in the brain among all examined WNV isolates, suggesting that Croc110 could replicate similarly as NY99 or Zmq16 in the brain (Fig. 4e). Taken together, these results indicate that the efficiency of virus replication in the periphery before brain entry differs between WNV isolates and Croc110 is less proliferative than NY99 or Zmq16 in the periphery.

## Discussion

The pathogenicity of WNV in crocodilians has been investigated via experimental infection with WNV strains that do not originate from crocodilians, such as NY99 (lineage 1a) and Kunjin virus (lineage 1b) [12,13]. There was no detailed information on WNV isolates derived from naturally infected crocodiles and their pathogenicity in mammals. In this study, Croc110, belonging to lineage 1a of WNVs circulating in Africa, the Middle East and Europe, was isolated from crocodiles with neurological signs. The results of virus propagation *in vitro* and in the brain after IC inoculation indicated that the efficiency of Croc110 replication in neuronal cells was comparable to that of the highly pathogenic strain NY99. However, when mice were peripherally infected with these WNV strains, the initial proliferation of Croc110 in peripheral tissues was lower than that of NY99, which might reduce its neuroinvasiveness and contribute to its low pathogenicity in mice.

On the contrary, mice infected with the lineage 2 WNV Zmq16, which was isolated from *Culex* mosquitoes in Zambia, displayed a comparable phenotype as those infected by the highly neuroinvasive NY99 strain. Zmq16 is genetically related to the South African isolates HS101_08, SPU116/89 and SA93/01, which were originally isolated from horse or human cases and they have been demonstrated to be highly pathogenic in mice [31,32]. Thus, in Zambia, WNV strains with different lineages and pathogenicity exist within the same country.

Previous studies on the pathogenicity of WNV in mice suggested that both lineage 1 and lineage 2 have highly and less neuroinvasive phenotypes and the pathogenicity is related to genetic differences but not to lineages or geographic distributions [31,32]. Several genetic determinants involved in WNV pathogenicity have been identified in the viral non-coding and protein-coding regions [17]. We noted that Croc110 possesses a serine residue at position 102 in NS4B protein, whereas other strains possess a cysteine residue (Table 1). It has been reported that the C102S substitution in NS4B attenuates NY99 proliferation at 41 °C and causes less neuroinvasive phenotypes in mice [33]. This C102S substitution in NS4B might be associated with the attenuated pathogenicity of Croc110 in mice. The high-temperature-sensitive phenotype of the C102S mutant also suggests that Croc110 replicates well in ectothermic crocodiles but not in mice. Further experiments are required to elucidate the factors associated with the differences in pathogenicity between crocodilians and mammals.

Our findings suggest that differences in pathogenicity between animal species should be considered in preventive measures against zoonotic WNV diseases and in the development of WNV vaccines for humans and crocodiles. Furthermore, continued monitoring for the infection status of each WNV strain, including its transmission route, is required to prevent West Nile fever epidemics in humans and crocodiles in Zambia.

## supplementary material

10.1099/jgv.0.002051 Uncited Fig. S1.

## Abbreviations

CPEs: cytopathic effects
DMEM: Dulbecco’s Modified Eagle’s Medium
dpi: days post-infection
IC: intracerebral
ID: intradermal
IP: intraperitoneal
MEM: Minimum Essential Medium
MGB: minor groove binder
RT-PCR: reverse transcription-polymerase chain reaction
RT-qPCR: Reverse transcription-quantitative polymerase chain reaction
TEM: transmission electron microscope
UTR: untranslated region
WNV: West Nile virus
