# Supplementary material for: African lineage 1a West Nile virus isolated from crocodiles exhibits low neuroinvasiveness in mice
Source: J Gen Virol. 2024 Nov 26;105(11):002051. doi: 10.1099/jgv.0.002051 (PMC11652736; doi:10.1099/jgv.0.002051)
Supplement: Uncited Fig. S1. [file jgv-105-02051-s001.pdf]

Fig. S1.

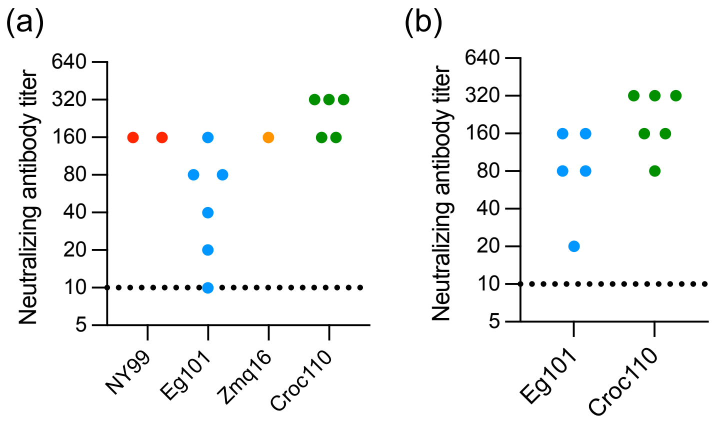

Fig. S1. The neutralizing antibody titer of surviving mice

Serum samples were collected from the surviving mice in Fig. 3a in one experiment. The neutralizing antibody titer was measured using Vero cells, and the dilution that inhibited CPEs by 50% was taken as the titer (a, intradermal inoculation; b, intraperitoneal inoculation). Samples with a 10-fold dilution or greater were considered WNV-positive.
